# Perceived stress and mobile phone addiction among college students: The roles of self-control and security

**DOI:** 10.3389/fpsyt.2022.1005062

**Published:** 2022-11-16

**Authors:** Anqi Zhang, Sicheng Xiong, Yu Peng, Yixin Zeng, Chengwei Zeng, Ying Yang, Bin Zhang

**Affiliations:** Department of Applied Psychology, Hunan University of Chinese Medicine, Changsha, China

**Keywords:** perceived stress, mobile phone addiction, self-control, security, college students

## Abstract

**Objective:**

According to the General Strain Theory, stress can lead to a range of problem behaviors. In the current study, we focused on the association between perceived stress and mobile phone addiction. We hypothesized that this association is mediated by low self-control and that the first path of the mediation is moderated by security.

**Methods:**

College students (*N* = 397; ages 16–21; 51.89% females) from a university in Hunan Province, China, were surveyed by cluster sampling method. The students completed the Smartphone Addiction Scale-Short Version (SAS-SV), the Depression Anxiety Stress Scale (DASS-21), the Self-Control Scale (SCS), and the Security Questionnaire (SQ) during regular class time. SPSS26.0 statistical software was used for descriptive statistics and Pearson correlation analyses, the SPSS macro PROCESS was used to test the mediating effects of self-control and the moderating role of security.

**Results:**

Mediation analysis showed that as expected, perceived stress was associated with lower self-control, which in turn was associated with a higher risk for mobile phone addiction. Also as expected, moderated mediation analysis indicated that the association between perceived stress and self-control was moderated by security. Specifically, the relationship between perceived stress and self-control was stronger for low security.

**Conclusion:**

This study provides useful insight into the understanding of how perceived stress increases the risk of mobile phone addiction. The results are consistent with the General Strain Theory and further indicate that concrete approaches are required for the prevention and intervention to reduce mobile phone addiction among college students.

## Introduction

Many people in the world rely on mobile phones and their increasingly integrated functions. China is no exception. According to the 48th Report of the China Internet Network Information Center, as of June, the number of Internet users in China reached 1.011 billion; of this group, the proportion that accessed the Internet to use mobile phones had increased from 98.6% at the end of 2018 to 99.6% at present (1). However, one concern that has attracted widespread attention is the possibility that some mobile phone users, especially younger users, spend too much time using their phones. According to a 2018 survey by the MyCOS Research Institute, Chinese college students use mobile phones for an average of more than 5 h a day, and 79% of college students use mobile phones during class (2). This extensive use of mobile phones has raised an even larger concern that overuse may lead to mobile phone addiction (3).

Mobile phone addiction is a form of behavioral addiction in which the individual’s physical, psychological and social functions are significantly impaired due to excessive use of mobile phones (4). Substantial research has consistently shown that mobile phone addiction has negative effects on physical, mental, and social functions. De-Sola et al. found that problematic cell phone use can lead to situations in which they lose control (5). Elhai et al. reported depression severity and anxiety severity were consistently related to problematic smartphone use (6). Likewise, shyness and attachment anxiety were positively associated with mobile phone addiction and were negatively associated with self-control (7). To develop effective prevention and remediation measures, it is critical to explore the inner mechanism of mobile phone addiction among college students.

Previous research has revealed that perceived stress is one of the crucial antecedents of mobile phone addiction (8–10). According to the General Strain Theory, many problematic behaviors result from negative experiences caused by various pressures or tensions (11). This theory is the most commonly used framework to explain perceived stress as a predictor of mobile phone addiction (12, 13). However, previous studies mostly focused on the direct relationship between perceived stress and mobile phone addiction (8); although a study has explored the underlying mechanisms, which have focused more on the negative perspective (14), there was a lack of attention to “how” perceived stress affects mobile phone addiction (mediating effect) from a positive perspective and under what circumstances it will have a stronger or weaker effect (moderating effect) on mobile phone addiction. With the development of positive psychology in recent years, the academic community has placed increasing emphasis on constructing and developing positive psychological factors in individuals. Therefore, the present study examined an underlying mediation mechanism (self-control) and moderating factor (security) in the association between perceived stress and mobile phone addiction from a positive psychological perspective. Based on the above analysis, we put forward the following hypothesis:

Hypothesis 1: Perceived stress is positively associated with mobile phone addiction.

### Self-control as a mediator

Self-control refers to the ability to resist inner desires and external temptations and adhere to expected goals (15). According to The Strength Model of Self-Control, the individual has a limited amount of energy for self-control over a certain period of time (16), and the energy in the energy pool is shared by activities in different fields, such as persistence, attention regulation, and emotion regulation (17, 18). The consumption of self-control energy in one field leads to the reduction of available energy in other fields (19). Previous research has shown that coping stress leads to poorer control over alcohol for recovering alcoholics (20).

Perceived stress is thought to reduce self-control resources in two ways (21). One possibility is that perceived stress generates negative emotions (22), and self-control resources have to be used to regulate these emotions. The second is that perceived stress triggers maladaptive cognitions such as rumination, and controlling rumination will significantly inhibit the ability of self-control (23). Therefore, perceived stress may trigger the consumption of psychological resources and weaken self-control.

Low self-control has been shown to have a positive association with mobile phone addiction (24, 25). Addictive behaviors arise mainly due to the individual’s weak self-control, which in turn leads to low self-regulation and the individual’s difficulty in suppressing the craving for addictive substances and causes addictive behaviors to occur (26). Therefore, a lower level of self-control may increase the risk of mobile phone addiction. We hypothesized that:

Hypothesis 2: Self-control mediates the relationship between perceived stress and mobile phone addiction.

### Security as a moderator

The mediation process by which perceived stress is associated with mobile phone addiction via low self-control may not be equally strong for all individuals. As a basic psychological need, security refers to having a sense of inner control in the face of stress, including the anticipation of possible future physical and psychological threats in the future, which is mainly expressed as a sense of certainty and control over the outside world (27).

First, previous studies have shown that security moderates the association between perceived stress and problematic psychological development, such as loneliness, depression, and personality development (28, 29). Second, security can buffer the relationship between perceived stress and problem behaviors, including aggression and suicidal ideation (30, 31). Mobile phone addiction is another problem behavior that may be predicted by perceived stress, with the association being stronger for those with low self-control. Specifically, compared with people with high security, those with low security are emotionally unstable, anxious, and interpersonally sensitive (27) and will turn their attention to the virtual world to seek emotional compensation and substitution when dealing with negative events (such as stress) (32). This suggests that low security may increase the possibility of mobile phone addiction.

Hypothesis 3: Security buffers the association between perceived stress and mobile phone addiction; specifically, the relationship between perceived stress and mobile phone addiction will be stronger in individuals with low security than those with high security.

In addition, compared to people with high security, those with low security may have more difficulty exerting self-control when coping with stress. That is, the mediating role of self-control in the association between perceived stress and mobile phone addiction should be stronger for people with low security. Gentzler et al. found that individuals with high security tend to enjoy their own positive experiences when faced with negative stimuli, whereas individuals with low security tend to repeatedly think about negative experiences (33), which in turn trigger negative emotions (34), and this inhibition of ruminative and regulation of negative emotions deplete individuals’ self-control resources (22, 23).

Hypothesis 4: Security moderates the first path of the mediation process. Specifically, the relationship between perceived stress and self-control will be stronger for participants with low security.

**FIGURE 1:**
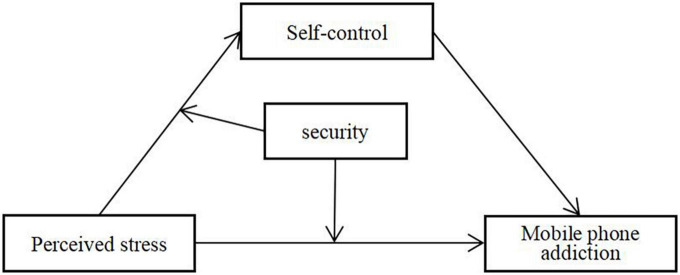
The moderated mediation model.

Thus, we developed and tested a moderated mediation model in which the association between perceived stress and mobile phone addiction is mediated by self-control and moderated by security (Figure 1).

## Materials and methods

### Participants and procedure

Participants were recruited from one university in a large city in Central China. A total of 425 freshmen and sophomores were selected by cluster sampling. After getting rid of the invalid questionnaires (questionnaires with lots of blanks or repeated answers, 397 participants were retained—the valid response rate was 93.4%. The age range of participants was 16–21 years (*M*_*age*_ = 18.30 years, *SD*_*age*_ = 0.86), 206 were females (51.89%), and 191 (48.11%) were males. Among them, 273 were freshmen (68.8%) and 124 were sophomores (31.2%).

### Measures

#### Short version of the smartphone addiction scale

The Chinese form (35) of the Short Version of the Smartphone Addiction Scale (SAS-SV) (36) was used to measure mobile phone addiction. The scale consists of 10 items (sample item: “I have missed planned studies or work because I was using my phone”). Participants rated each item on a six-point scale (1 = completely disagree, 6 = completely agree), with higher scores indicating a higher level of mobile phone addiction. Cronbach’s α in this study was 0.87.

#### Depression anxiety stress scale

The stress subscale from the Chinese version (37) of the Depression-Anxiety-Stress Scale (DASS-21) (38) was used to assess the symptoms of stress individuals experience. The subscale of stress consists of 7 items (sample item: “I find it hard to keep myself quiet”) rated on a four-point scale (1 = disagree to 4 = completely agree), with higher scores indicating a higher level of perceived stress. In this study, Cronbach’s α for this scale was 0.87.

#### Self-control scale

The Chinese version (39) of the Self-Control Scale (SCS) (15) was used to assess degree of self-control. This scale has 13 items to assess two dimensions of self-control, namely impulse control (eight items; e.g., “I have a hard time dropping bad habits”) and self-discipline (five items; e.g., “I am good at rejecting things that are not good for me”). Participants rated each item on a five-point scale (1 = completely agree to 5 = completely disagree). Higher scores indicate a higher level of self-control. Cronbach’s α in this study was 0.86.

#### Security questionnaire

Security was measured by the Security Questionnaire (SQ) (40). The scale has 16 items to assess two dimensions of security, namely interpersonal security (eight items; e.g., “I’ve never dared to volunteer my opinion”) and certainty control (eight items; e.g., “I feel that life is always full of uncertainty and unpredictability”). The scale uses a 4-point scale (1 = completely consistent, 5 = completely inconsistent). The higher the score, the higher the individual’s sense of security. In this study, Cronbach’s α for this scale was 0.93.

### Procedure

Psychology graduate students were trained as research assistants to distribute questionnaires in each class. They gave instructions and reminded the volunteers that participation was voluntary. It took about 20 min to complete the questionnaires and all participants were rewarded with an online shopping voucher which is worth five yuan after participation. All materials and procedures were reviewed and approved by the Ethics Committee of Hunan University of Chinese Medicine, and all participants and their legal guardians involved in the study provided written informed consent according to the ethical guidelines set forth in the Declaration of Helsinki.

### Data analysis

The statistical analyses were conducted in SPSS 26.0. First, descriptive statistics and Pearson correlation analyses were conducted. The second step was to test the mediation model and moderated mediation model using the SPSS macro PROCESS version 4.0 (Model 4 and Model 8) developed by Hayes (41). Because previous research indicated that mobile phone addiction may vary based on gender and age (42), these were included as covariates in the present study. Harman’s single-factor test was used to investigate common method bias. The result of this test showed that the variation in the first factor explained 23.91% of the total variance. This value was less than the cutoff of 40% (43), indicating that common method bias was not a substantial problem.

## Results

### Descriptive statistics

Descriptive statistics and correlations were computed for all variables and are reported in Table 1. Correlation analyses revealed that perceived stress was significantly and positively correlated with mobile phone addiction, and was significantly and negatively correlated with self-control and security. Mobile phone addiction was significantly and negatively correlated with self-control and security. Self-control was significantly and positively correlated with security.

**TABLE 1:** Descriptive statistics and correlations among variables.

Variable	M ± *SD*	1	2	3	4	5	6
1. Perceived stress	10.91 ± 3.51	1					
2. Mobile phone addiction	35.62 ± 8.39	0.30**	1				
3. Self-control	41.09 ± 6.29	−0.38**	−0.55**	1			
4. Security	53.98 ± 11.26	−0.35**	−0.24**	0.35**	1		
5. Gender	0.52 ± 0.41	0.05	0.13*	–0.08	–0.07	1	
6. Age	18.30 ± 0.86	0.03	–0.03	0.02	0.03	–0.08	1

*N* = 397. *p < 0.05. **p < 0.01. Male = 0, Female = 1.

### Testing for the mediation model

Model 4 from the SPSS macro PROCESS was used to test for mediation. After controlling for gender and age, perceived stress was positively associated with mobile phone addiction (β = 0.30, *p* < 0.01). Table 2 shows that the direct predictive effect of perceived stress was still significant (β = 0.11, *p* < 0.05) when self-control was included as a mediator. Perceived stress was significantly negatively associated with self-control (β = –0.38, *p* < 0.01). Self-control was significantly negatively associated with mobile phone addiction (β = –0.50, *p* < 0.01). Table 3 shows the bootstrap 95% confidence interval around each effect. An effect is considered significant when the confidence interval does not include 0. The mediation effect accounted for 63.33% of the total effect.

**TABLE 2:** Summary table of mediation effect analysis.

Predictor	Self-control			Mobile phone addiction		
	β	SE	95% CI	β	SE	95% CI
Constant	–0.46	1.03	[–2.47, 1.56]	–0.06	0.91	[–1.85, 1.73]
Age	0.03	0.06	[–0.08, 0.14]	–0.01	0.05	[–0.10, 0.09]
Gender	–0.13	0.12	[–0.36, 0.09]	0.20	0.10	[–0.01, 0.40]
Perceived stress	−0.38**	0.05	[–0.47, -0.29]	0.11*	0.05	[0.02, 0.20]
Self-control				−0.50**	0.50	[–0.59, –0.41]
*R* ^2^	0.15			0.32		
*F*	22.54**			45.43**		

*N* = 397. Bootstrap sample size = 5,000. CI, confidence interval. B = standardized coefficient. *p < 0.05. **p < 0.01. Male = 0, Female = 1.

**TABLE 3:** Bootstrapping indirect effect and 95% confidence interval (CI) for the mediation model.

	Estimated effect	SE	95% CI	Ratio to total effect
Total effect	0.30	0.05	[0.21, 0.40]	
Direct effect	0.11	0.05	[0.02, 0.20]	36.67%
Indirect effect	0.19	0.04	[0.10, 0.28]	63.33%

*N* = 397. Bootstrap sample size = 5,000. CI, confidence interval.

### Testing for the moderated mediation model

Model 8 from the SPSS macro PROCESS was used to test the proposed moderated mediation model with self-control as mediator and security as moderator, after controlling for gender and age. Table 4 shows that after adding security into the model, the interaction between perceived stress and security has a significant predictive effect on self-control (β = 0.16, *p* < 0.01) but not on mobile phone addiction (β = –0.03, *p* > 0.05). Thus, as expected, security moderated the first path of the mediating process (perceived stress → self-control). Contrary to our hypothesis, security did not moderate the direct process (perceived stress → mobile phone addiction).

**TABLE 4:** Summary of moderated mediation effect analysis.

Predictor	Self-control			Mobile phone addiction		
	β	SE	95% CI	β	SE	95% CI
Constant	–0.95	0.97	[–2.86, 0.95]	–0.05	0.92	[–1.87, 1.76]
Age	0.06	0.05	[–0.05, 0.16]	–0.01	0.05	[–0.10, 0.09]
Gender	–0.07	0.11	[–0.28, 0.14]	0.19	0.10	[–0.01, 0.39]
Perceived stress	−0.25**	0.05	[–0.34, -0.16]	0.10*	0.05	[0.01, 0.20]
Self-control				−0.49**	0.05	[–0.58, -0.39]
Security	0.25**	0.05	[0.16, 0.35]	–0.03	0.05	[–0.12, 0.07]
Perceived stress × security	0.16**	0.03	[0.10, 0.22]	–0.01	0.03	[–0.06, 0.05]
*R* ^2^	0.26			0.32		
*F*	26.92**			30.21**		

*N* = 397. Bootstrap sample size = 5,000. CI, confidence interval. B = standardized coefficient. *p < 0.05. **p < 0.01. Male = 0, Female = 1.

**FIGURE 2:**
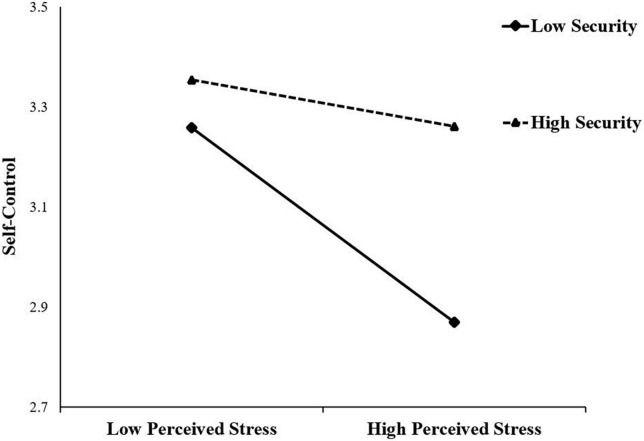
Security moderated the association between perceived stress and self-control.

Simple slopes analysis was used to interpret the interaction effect. Figure 2 shows the association between perceived stress and self-control at two levels of security (low level, 1 SD below the mean; high level, 1 SD above the mean). For individuals with low security, perceived stress was significantly negatively associated with self-control (simple slope = –0.39, *p* < 0.001); for individuals with high security, the predictive effect was not significant (simple slope = –0.09, *p* > 0.05). These analyses indicated that the predictive effect of perceived stress on self-control decreases with increased security.

Security also moderated the overall indirect effect (perceived stress → self-control → mobile phone addiction) with the index of moderated mediation = –0.08, Boot SE = 0.03, and 95% Confidence Interval [–0.13, –0.01]. For individuals with a high level of security, the indirect effect of perceived stress on mobile phone addiction through self-control was not significant, with effect = –0.42, Boot SE = 0.64 and 95% Confidence Interval [–1.15, 1.52]. For individuals with low security, the indirect effect of perceived stress on mobile phone addiction through self-control was significant, with effect = –0.14, Boot SE = 0.10, and 95% Confidence Interval [–0.40, –0.01].

To further verify the validity of the results, this study used a structural equation model to assess the mediating effect of self-control between perceived stress and mobile phone addiction. In addition to constructing the hypothesized model, other competitive models were constructed to test the superiority of the model. Table 5 shows that the observed data fit the hypothesized model best (χ^2^*/df* = 3.924, CFI = 0.939, TLI = 0.888, RMSEA = 0.086, SRMR = 0.041).

**TABLE 5:** Hypothesized model and competitive model fitting parameters.

Model	Description	χ^2^/*df*	RMSEA	CFI	TLI	SRMR
Hypothesized model	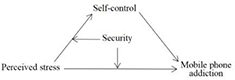	3.924	0.086	0.939	0.888	0.041
Competitive model 1	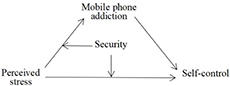	6.312	0.116	0.872	0.797	0.079
Competitive model 2	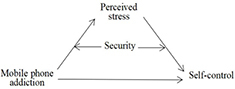	5.864	0.111	0.855	0.710	0.080
Competitive model 3	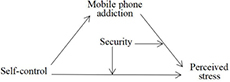	4.454	0.093	0.922	0.883	0.081
Competitive model 4	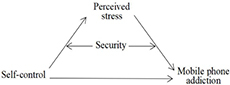	7.378	0.127	0.885	0.784	0.064
Competitive model 5	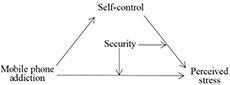	4.299	0.091	0.925	0.893	0.082

## Discussion

The general question in this study was whether college students who perceive high stress are also at higher risk of mobile phone addiction. We established this connection, consistent with other research (44), which fits with the General Strain Theory that individuals typically develop problem behaviors to alleviate stressful feelings when exposed to stressful events (11, 12). We were then able to contribute to the literature by examining other positive psychological factors that might explain or contribute to this association from a positive psychological perspective. As expected, college students who perceived higher stress tended to show lower self-control, and in turn, more behaviors associated with mobile phone addiction. Also as expected, this process was stronger for those students who had a low sense of security. The obtained findings not only contribute to the understanding of how perceived stress was associated with mobile phone addiction but also provide a broad perspective toward the prevention and intervention of mobile phone addiction among college students.

### The mediating role of self-control

Low self-control mediated the relationship between perceived stress and mobile phone addiction, which verifies Hypothesis 2 of this study and supports the Strength Model of Self-Control. Both parts of this process (perceived stress → low self-control) and (low self-control → mobile phone addiction) can be conceptualized in terms of demands on psychological resources. With regard to the first path, stress may trigger negative emotion and negative cognition when they feel stressed (22). The regulation of these feelings and thoughts occupies self-control resources, resulting in a shortage of corresponding resources in other fields. With lower self-control resources available, there is a decline in self-control ability (21). With regard to the second path, individuals in a low self-control state lack the psychological resources to resist acting impulsively. Low self-control was associated with mobile phone addiction (45), and people who show behaviors associated with mobile phone addiction have difficulty inhibiting the impulse to use mobile phones (46).

The Dual Systems Model of Self-Control can help us conceptualize this mediated effect in terms of a lack of resources. According to this model, there are two main systems of individual executive function: the rational system and the impulse system. When resources are sufficient and the main activities can be carried out smoothly, the rational system occupies the dominant position and guides the individual to show self-control. In this state, the person can resist immediate satisfaction, think carefully, and make thoughtful decisions. However, because self-control starts to drain resources, the main activities cannot be carried out. The impulse system is then engaged to encourage intuitive, heuristic processing to meet the current ideas and needs (47). As a mobile device that combines utility and entertainment, mobile phones are used by a wide range of users to satisfy their various needs, which further increases the risk of mobile phone addiction.

### The moderating role of security

It is found for the first time that security moderates the first path of the mediation process (perceived stress → self-control), which verifies Hypothesis 4 of this study. Indeed, among college students with higher security, there was a weaker association between perceived stress and self-control; compared to students with lower security, those with high security still maintained self-control while enduring higher perceived stress. Individuals with low security are more afraid of losing control of the outside world and the recurrence of stress events. Therefore, they fall into the pain of negative emotions (48), and the resources for self-control are consumed (17). For people with low security, the association between perceived stress and self-control will be intensified, the mediating effect of self-control in the association between perceived stress and mobile phone addiction is also stronger.

By contrast, students with high security may still trust the certainty of the external environment and their own control in the face of stress. These students are better able to handle perceived stress due to their belief that the external environment is controllable. Therefore, they are less likely to be immersed in negative emotions and maladaptive cognitions (33), self-control resources are more abundant, perceived stress is a weaker predictor of self-control, and the mediating effect of self-control in the association between perceived stress and mobile phone addiction will not be significant.

The relationship between perceived stress and mobile phone addiction was not moderated by security, Hypothesis 3 has not been verified. This finding was contrary to expectations. Regardless of the level of individual security, the positive relationship between perceived stress and mobile phone addiction appears to be stable. One reason could be that the data were collected during the COVID-19 pandemic of this study, the perceived stress state of college students during this period (10.91 ± 3.51) was significantly higher than it was pre-pandemic (6.12 ± 5.54) (37), *t*(2,174) = 16.51, *p* < 0.001, Cohen’s *d* = 0.92. In addition, according to the cut-off point of 32 for mobile phone addiction on the Short Version of the Smartphone Addiction Scale (49), the prevalence of mobile phone addiction among college students in this study was 68.01%, higher than the results of recent studies (22.8—40.6%) based on the same measure (50–52). Consistent with previous studies, researchers have identified that the epidemic had a negative impact on both the mental health and lifestyle of individuals, such as stress (53), panic, depression (54), and unhealthy diet behaviors, poorer sleep quality, physical inactivity (55–57), and smartphone addiction (58). As a result, the levels of perceived stress and mobile phone addiction remain high and difficult to alleviate, which suggest that in today’s electronic information age, mobile phones have become the most convenient and effective tool for emotional contact, learning and communication, and online entertainment for college students in response to major public health events, and thus their frequency of smartphone use has increased significantly, which also greatly increases the risk of mobile phone addiction, and therefore the relationship between perceived stress and mobile phone addiction may be very stable during this period, regardless of their specific level of security. Future research can be further discussed in this regard.

### Implications for theory and practice

Based on the General Strain Theory and the Strength Model of Self-Control, this study reveals a specific mechanism by which perceived stress is related to mobile phone addiction, enriches and develops relevant theories, and provides practical guidance for reducing the incidence of this problematic behavior. First, as a predictor of problematic behavior, perceived stress is an important “fuse” for the occurrence of mobile phone addiction. Parents and educators should pay full attention to the student’s feelings under stress and create a relatively less stressful external environment to promote adaptive functioning.

Second, training of individual self-control ability may weaken the “bridge” between perceived stress and mobile phone addiction. Finally, as a basic psychological need (27), security appears to be a buffer against the effects of perceived stress on self-control. Therefore, parents and schools should create a safe and trusted growth environment for children and help them establish an appropriate security from childhood, so as to resist the occurrence and development of problematic behavior.

### Limitations and prospects

There are some shortcomings in this study, which provide directions for further research. First, although this study constructs a moderated mediation model based on theories as well as excluding other competitive models, it is still cross-sectional. Therefore, follow-up studies can use a longitudinal design to investigate the relationships among perceived stress, self-control, and mobile phone addiction. Second, the participants were freshmen and sophomores in a university in Hunan Province. Therefore, whether the research results can be extended to other populations remains to be examined. Future research should consider investigating in multiple samples. Third, even though the self-report data were anonymous, there may still be an effect of social desirability bias. A variety of evaluation methods can be considered (such as combining the evaluations made by parents, peers, and teachers; or using smartphone software to record how often and for hold long phones are being used) to make up for the limitations of self-report data. Fourth, mobile phone addiction may have comorbidity with other psychological symptoms (e.g., depression, anxiety, etc.), and these variables may be controlled or explored as comorbidity in future studies. Last, the data were collected during the epidemic period, resulting in high levels of some variables. The results of this study mainly focus on the formation mechanism of mobile phone addiction among college students under the current normalization of epidemic prevention and control, so whether they can be extended to young adults in non-epidemic areas needs further validation in the future.

## Conclusion

Perceived stress positively predicted college students’ mobile phone addiction. Self-control mediated the relationship between perceived stress and mobile phone addiction. Moreover, security moderated the first path of the mediation processes. Compared with students who had lower security, those with higher security demonstrated a weaker association between perceived stress and self-control.

## Data availability statement

The raw data supporting the conclusions of this article will be made available by the authors, without undue reservation.

## Ethics statement

All materials and procedures were reviewed and approved by the Ethics Committee of Hunan University of Chinese Medicine, and all participants and their legal guardians involved in the study provided written informed consent to participate according to the ethical guidelines set forth in the Declaration of Helsinki.

## Author contributions

BZ and SX conceived the idea for the study. BZ, SX, YP, YZ, CZ, YY, and AZ obtained the data. AZ, CZ, and YP performed the data analyses. AZ wrote the manuscript with the participation of other authors. All authors contributed to the article and approved the submitted version.
